# Bladder Recovery by Stem Cell Based Cell Therapy in the Bladder Dysfunction Induced by Spinal Cord Injury: Systematic Review and Meta-Analysis

**DOI:** 10.1371/journal.pone.0113491

**Published:** 2015-03-17

**Authors:** Jae Heon Kim, Sung Ryul Shim, Seung Whan Doo, Won Jae Yang, Byung Wook Yoo, Joyce Mary Kim, Young Myoung Ko, Eun Seop Song, Ik Sung Lim, Hong Jun Lee, Yun Seob Song

**Affiliations:** 1 Department of Urology, Soonchunyang University Hospital, Soonchunhyang University School of Medicine, Seoul, Korea; 2 Institute for clinical molecular biology research, Soonchunyang University Hospital, Soonchunhyang University School of Medicine, Seoul, Korea; 3 Department of Family Medicine, Soonchunhyang University School of Medicine, Seoul, Korea; 4 International Clinic Center, Soonchunhyang University Hospital, Seoul, Korea; 5 Department of Industrial and Management Engineering, Pohang University of Science and Technology, Pohang, Korea; 6 Department of Obstetrics and Gynecology, Inha University School of Medicine, Incheon, Korea; 7 Department of Industrial Management and Engineering, Namseoul University College of Engineering, Cheonan, Korea; 8 Medical Research Institute, Chung-Ang University College of Medicine, Seoul, Korea; Medical University of South Carolina, UNITED STATES

## Abstract

**Background:**

Bladder dysfunction induced by spinal cord injury (SCI) can become problematic and severely impair the quality of life. Preclinical studies of spinal cord injury have largely focused on the recovery of limb function while neglecting to investigate bladder recovery.

**Objective:**

The present study was performed to investigate and review the effect of stem cell-based cell therapy on bladder recovery in SCI.

**Methods:**

We conducted a meta-analysis of urodynamic findings of experimental trials that included studies of stem cell-based cell therapy in SCI. Relevant studies were searched using MEDLINE, EMBASE and Cochrane Library (January 1990 - December 2012). Final inclusion was determined by a urodynamic study involving detailed numerical values. Urodynamic parameters for analysis included voiding pressure, residual urine, bladder capacity and non-voiding contraction (NVC). Meta-analysis of the data, including findings from urodynamic studies, was performed using the Mantel-Haenszel method.

**Results:**

A total of eight studies were included with a sample size of 224 subjects. The studies were divided into different subgroups by different models of SCI. After a stem cell-based cell therapy, voiding pressure (-6.35, *p* <0.00001, I^2^ = 77%), NVC (-3.58, *p* <0.00001, I^2^ = 82%), residual urine (-024, *p* = 0.004, I^2^ = 95%) showed overall significant improvement. Bladder capacity showed improvement after treatment only in the transection type (-0.23, *p* = 0.0002, I^2^ = 0%).

**Conclusion:**

After stem cell-based cell therapy in SCI, partial bladder recovery including improvement of voiding pressure, NVC, and residual urine was demonstrated. Additional studies are needed to confirm the detailed mechanism and to obtain an ideal treatment strategy for bladder recovery.

## Introduction

Worldwide, there are more than 130,000 new cases of traumatic spinal cord injury (SCI) annually, which is estimated to affect approximately 2.5 million people [1,2]. SCI affects people of all ages and the present lack of curative treatments result in life-long functional impairments that manifest as major physical disabilities [1]. Any recovery from functional loss that can occur depends on the level, nature and severity of the injury [3]. Severe injuries yield very poor outcomes and recovery is limited. Consequently, patients suffer from chronic paralysis and autonomic dysfunctions in the body segments below the site of injury [3]. In addition, SCI leads to disabling secondary complications, such as spasticity, bowel and bladder dysfunction, and development of chronic neuropathic pain [4].

Among the secondary complications caused by SCI, patients suffer from urological complications [5] that include severe lower urinary tract dysfunctions including overactive bladder and urinary retention, which result in increased bladder thickness and fibrosis [6,7]. The urinary bladder wall accumulates connective tissue and becomes fibrotic following SCI, which may adversely affect smooth muscle function and the micturition function resulting in a low compliance bladder with low capacity and high threshold pressure.

Although the significance of a neurogenic bladder caused by SCI has been documented in clinical studies associated with urinary tract infection and chronic renal failure [8], less focus has been directed to preclinical SCI studies, especially stem cell-based cell therapy. Most SCI studies with stem cell-based cell therapy have focused mainly on the motor limb function and sensory recovery. Impaired quality of life (QoL) from loss of limb function likely exceeds that of bladder dysfunction in the short term. However, in the long term, bladder dysfunction can become problematic and impair severely QoL [9]. Despite these concerns and obstacles, research on bladder recovery represents a promising area of research in neurourology. To date, similar to preclinical research, clinical trials have focused mainly on the recovery of motor and sensory function of limbs. Thus, limited evidence regarding bladder recovery exists [10,11].

In the present study, we investigated bladder recovery using stem cell-based cell therapy in bladder dysfunction induced by SCI employing meta-analysis of urodynamic findings in preclinical studies and highlighted the current status of stem cell-based cell therapy of bladder dysfunction in SCI with a systematic review.

## Materials and Methods

### Study design

A meta-analysis and systematic review were conducted according to predefined guidelines provided by the Cochrane Collaboration (2008).

### Study selection

A comprehensive, multi-database electronic literature search was done to identify relevant research articles published between December 1990 and December 2012 involving varied SCI preclinical studies including stem cell-based cell therapy. The electronic search included the PubMed, Embase and Cochrane Library databases without language restrictions. The Medical Subject Headings (MeSH) and Emtree keywords included ‘‘spinal cord injuries”, “spinal injuries”, ‘‘stem cells’,’ ‘‘cells, cultured”, “stem cell transplantation” and “Rat”.

The studies were included if the described experiments included spinal surgery; the described interventions included administration of stem cell or other progenitor cells; the reported outcomes included urodynamic findings like voiding pressure, residual urine, non-voiding contraction (NVC) and bladder capacity; and rats were the experimental animal. The articles were excluded if the studies were unable to acquire full urodynamics data, involved non-randomized controlled animal trial of low quality, or involved combined treatment with medications.

### Data collection and analysis

The initial screening with electronic databases to identify potential studies for inclusion based on title and abstract information was performed independently by two of the authors (Jae Heon Kim and Sung Ryul Shim). In cases of insufficient data, both authors reviewed the full text of the article for information and clarification. Final inclusion was determined by the senior authors (Hong Jun Lee and Yun Seob Song). References and data for each included study were carefully cross-checked to ensure no overlapping data was presented and to maintain the meta-analysis integrity.

### Assessment of methodological quality

The include studies assessed for risk of bias by the GRADE Working Group and a managing reviewer according to the Cochrane guidelines. The judgment of every item was low risk, unclear or high risk. Any disagreement regarding eligibility during the extraction was discussed and resolved. Six assessed items were random sequence generation, allocation concealment, blinding of outcome assessment, incomplete outcome data, selective reporting, and other bias.

### Meta-analysis of urodynamic findings

Meta-analysis of the data including findings from urodynamic studies was performed using the Mantel-Haenszel method with Review Manager software (RevMan version 5.0; Cochrane Collaboration, Germany) and Comprehensive Meta-Analysis version 2.2 software (Biostat, Englewood, NJ, USA). All variables were continuous data, such as voiding pressure, residual urine, NVC and bladder capacity. Mean ± standard deviation was used to calculate the weighted mean difference (WMD) and 95% confidence interval (CI).

### Assessment of heterogeneity

Statistical heterogeneity was assessed using the I^2^ value and the result of the chi-squared test. A *p* <0.1 and I^2^ >50% were considered suggestive of statistical heterogeneity, prompting a random effects modeling estimate. However, a non-significant chi-squared test result (*p* ≥0.1 and I^2^ ≤50%) indicated a lack of evidence for heterogeneity, but did not necessarily imply homogeneity, because there may have been insufficient power to detect heterogeneity.

### Assessment of potential publication bias

Results of the comprehensive meta-analysis to examine any potential publication bias in the studies are shown as Funnel plots.

## Results

### Inclusion of studies

**Fig 1:**
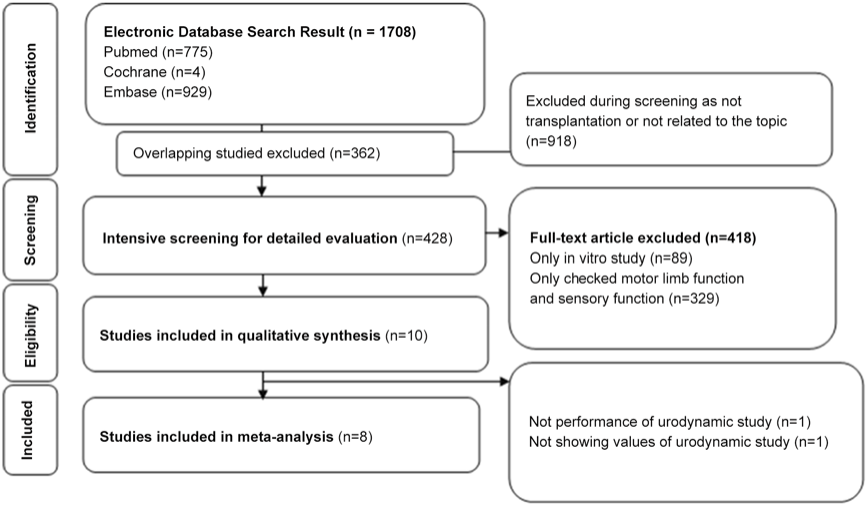
Method for study search and selection for inclusion.

The initial search identified 1708 articles from the electronic databases (929 from Embase, 775 from Pubmed, four from Cochrane). After exclusion of 362 studies containing overlapping data or appearing in more than one database, and after screening the titles and abstracts, 918 studies that did not meet the inclusion criteria were further excluded. After intensive screening for detailed evaluation of the remaining 428 studies, 10 studies were eligible. Of these, two were excluded due to lack of specific urodynamic study information. Finally, eight studies that met all inclusion criteria were included. The eight studies consisted of 222subjects (110 experimental and 112 controls). A detailed flow chart for selection is shown in Fig. 1. A systematic review of the 10 studies was conducted on the detailed experimental differences and expected mechanisms (Tables 1 and 2). In five studies [12–16], two different treatment subgroups were included. Therefore, the meta-analysis comprised a total of 13 trials (Table 3).

**Table 1:** Details of studies investigating stem cell-based cell therapy in spinal cord injury and bladder dysfunction.

Author	Injured level	Injury mechanism	Admitted cell	Route	Remarks	Expected mechanism	Graft survival confirmation
**Hu, 2012[17]**	T8–9	Transection	BMSC, Bone marrow stromal cell	Intravenous	External urethral sphincter activity was checked, Cooridnation between bladder activity and EUS was checked	No sparing of descending modulatory pathway. Reorganization of synaptic connections. Formation of new pathways of voiding reflex	Immunohistochemistry of BrdU reactive cell
**Jin, 2011[12]**	T9–10	Contusion	GRP, GDA	Direct injection	Daily records of bladder size and urine color, Human GRP and GDA did not promote axon growth	Neuroprotection, axonal sprouting, regeneration or myelination	HuNA (+) Immunofluorescence
**Park, 2010[15]**	T8–9	Contusion	Human MSC	Direct injection	BDNF and NT-3 levels were checked by ELISA and RT-PCR at 28 and 56 days after MSC transplantation. No bladder recovery	Transplanted MSCs could reduce inflammatory process	Immunohistochemistry of anti-human nucleus antibody
**Mitsui, 2011 [13]**	T8–9	Contusion	NRP/GRP, AMPA/kinase blocker (NBQX)	Direct injection	Combined treatment using NRP/GRP and NBQX	Combined treatment have a potential of protection of host tissue and reduction of secondary injury	Not checked
**Erdogan, 2013[43]**	T8–9	Transection, incomplete	Human umbilical cord MSC	Direct injection	Masson trichome stain used to demonstrate bladder morphology	Bladder structure change by reducing inflammation. Lamina propia layer change demonstrates reduced inflammation	Not checked
**Telmeltas, 2009[16]**	T9–10	Hemisection	NRP/GRP, BMSC	Direct injection	Camparison of transplantation using NRP/GRP and BMSC	Neurogenesis by the capacity to grow new neurons from neural stem/progenitor cells	Direct count of cells
**Mitsui. 2005 [18]**	T8–9	Contusion	Fb-BDNF, Fb-NT3	Direct injection	Genetically modified fibroblast	Partial bladder recovery is mediated by the greater density of some descending fibers and decreased density of small caliber primary afferent projections by BDNF or NT3. Tissue sparing throughout the white matter and differentiation into neurons and glia	Direct cell confirmation by Nissl myelin staining. mmunoreactivity for RT-97
**Neuhuber, 2008 [44]**	T8–9	Contusion	NPC, Neural progenitor cell	Direct injection or lumbar puncture graft	Different route and method were compared. Direct injection vs lumbar puncture graft. Lumbar puncture graft showed localization at the dorsal aspect rather than entire injury site, which results in partial recovery of motor function		Direct cell confirmation by Nissl myelin staining. Alkaline phosphatase histochemistry
**Mitsui, 2005 [14]**	T8–9	Contusion	NRP/GRP	Direct injection	Examined the alpha-1A adrenergic antagonist effects. Tamsulosin showed positive effect in voiding pressure	Local protection provided by NRP/GRP results in increased sparing/sprouting of descending pathways. Modification of lumbosacral circuitry	Direct cell confirmation by Nissl myelin staining. Alkaline phosphatase histochemistry
**Mitsui, 2003 [19]**	T8–9	Contusion	EG6 immortalized neural stem cell	Direct injection	Observed difference in the degree of functional recovery between detrusor and external urethral sphincter: Functional recovery of somatic nerves is more easily accomplished by transplanted EG6 cells	Transplanted EG6 cells might differentiate and promote regeneration of injured spinal cord	Confirmation with BrdU immunohistochemistry

GRP, glial-restricted precursor; GDA, derived astrocytes; MSC, mesenchymal stem cell; BDNF: brain derived neurotrophic factor; NRP, neuronal-restricted precursor; NBQX, 2,3-dihydroxy-6-nitro-7-sulfamoyl-benzo(f)quinoxaline; BMSC, bone marrow stromal cell; NT-3,neurotrophin-3; Fb, fibroblast; NPC

**Table 2:** Study details including defense mechanisms, stability and plasticity of stem cells in stem cell-based cell therapy for SCI and bladder dysfunction.

Author	Migration	Host effects: spared host tissue, cyst and scar formation	Plasticity or projections	Differentiation	Functional recovery	Sensory recovery
**Hu 2012[17]**	Yes	Not checked	L3–4	Not checked	Not checked	Not checked
**Jin, 2011[12]**	Yes, long distance	Reduced cyst and scar formation. Reduced CSPG expression. Reduced glial scar formation	L6-S1, GRP Group showed reduced CGRP sprouting, GDA group showed higher 5-HT immunoreactivity	Differentiated into glial cells	No significant recovery in BBB test and grid test	Pain perception improved Thermal sensitivity (heat test). Mechanical sensitivity (Von Frey test)
**Park, 2010[15]**	No checked	Not checked	BDNF and NT-3 levels showed no difference at thoracic and lumbar vertebrae L5-S1	Not differentiated	Limited recovery in chronic state in BBB test, coupling score, and ladder score	Not checked
**Mitsui, 2011[13]**	No checked	Reduced cyst and cavity formation	5HT positive fibers showed higher density in both DL and DH. CGRP positive fibers in small diameter dorsal root afferents	Not checked	Recovery in BBB test	Not checked
**Erdogan, 2013[43]**	Not checked	Not checked	Not checked	Not checked	Not checked	Not checked
**Telmeltas, 2009 [16]**	Not checked	Not checked	T9–10, E-NCAM (+), A2B5(+)	Neuronal differentiation	Not checked	Not checked
**Mitsui. 2005 [18]**	Confirmed by Nissl-myelin staining	No difference in astrocytic scar formation	L6-S1, Afferent pathways using CGRP and VR-1. Suppression in transplanted group. No change in synaptic density. Descending pathways using 5HT, CRF, DβH. Postive for markers for neurons (MAP-2). Partially preserved or stimulated sprouting. No axonal growth by GAP-43	Partially neuronal regeneration. No axonal growth	No significant recovery in BBB test and recovery in grid test	No change in thermal sensitivity test
**Neuhuber, 2008 [44]**	Some graft derived glial cells migrated away from the graft tissues Confirmed by Nissl myelin staining	Both group showed reduction in injury size and tissue sparing	L4–5, Oligodendrocytes (RIP) and astrocytes (GFAP)	Presence of neuronal and glial phenotypes. Neuronal differentiation	Intermediate recovery in BBB test	Not checked
**Mitsui, 2005 [14]**	Confirmed by Nissl myelin staining	Spared host tissue and cyst developed fewer	L6-S1, Afferent pathways: no differences in CGRP, VR-1, GAP43. Synaptophysin projections. NRP/GRP transplants inhibit dorsal root sprouting: CRF and DβH were diminished but lesser extent in transplant group. Sprouting or sparing of these pathways.	Neuronal differentiation Axonal neurofilaments with RT 97 antibody astrocyte differentiation with GFAP	Greater recovery of hindlimb function but similar results in grid test	Improvement in the thermal stimulus
**Mitsui, 2003[19]**	BrdU, Migration into white matter	Not checked	Not checked	Not checked	Not checked	Not checked

CSPG, Chondroitin sulfate proteoglycan;, neuronal-restricted precursor; VR-1,vanilloid receptor type 1; CGRP, calcitonin gene-related peptide; DL, Dorsolateral nucleus; DH, Dorsal horn; BBB, Basso, Beattie, Bresnahan; 5-HT, serotonin; DβH, dopamine-β-hydroxylase; CRF, corticotropin releasing factor; GFAP, glial fibrillary acidic protein; GRP, glial-restricted precursor; GAP43, growth-associated protein 43

**Table 3:** Urodynamic study results of the eight enrolled studies.

Author	Admitted time	Cystometry time	Cystometric findings	Sham	Control	Transplanted group
**Hu 2012 [17]**	9 days after injury	28 days after transplantation		N = 6	N = 8	N = 8
			Voiding pressure	26.35±4.18	40.74±3.97	33.82±3.79*
			Residual urine (ml)	0.1±0.08	1.53±0.68	0.89±0.17*
			Bladder capacity (ml)	0.35±0.14	1.57±0.68	1.20±0.22
			Voiding efficiency (%)	70.38±10.10	2.78±1.46	26.25±6.93*
			NVC	0	9.67±3.83	2.13±1.13*
**Jin, 2011 [12]**	9 days after injury	8 weeks after transplantation		N = 4	N = 10	N = 9, Transplanted CRP
			Voiding pressure	15.58±2.58	42.32±4.07	36.21±1.72
			Residual urine	0±0	0.12±0.08	0.42±0.13
			Bladder capacity	0.33±0.03	0.60±0.20	1.04±0.10*
			NVC	0.25±0.15	16.36±5.57	6.25±0.79*
			Bladder weight: body weight ratio (g)	0.61±0.02	1.3±0.07	1.43±0.09
				N = 4	N = 10	N = 6, Transplanted GDA
			Voiding pressure	15.58±2.58	42.32±4.07	37.21±2.04
			Residual urine	0±0	0.12±0.08	0.13±0.08
			Bladder capacity	0.33±0.03	0.60±0.20	0.53±0.05
			NVC	0.25±0.15	16.36±5.57	19.25±1.70
			Bladder weight: body weight ratio (g)	0.61±0.02	1.3±0.07	1.38±0.08
**Park, 2010 [15]**	9 days after injury	28 days and 56 days		N = 7	N = 11	N = 9, Transplanted (28 days)
			Voiding pressure	9.21±5.30	9.60±4.2	9.70±4.11
			NVC (%)	43	36	44
			Voiding frequency (time/min)	0.80±0.09	0.82±0.16	0.76±0.32
			Bladder volume (mm^3^)	1345.25±1206.27	1263.75±838.05	1279.57±1310.69
				N = 7	N = 9	N = 6, Transplanted (56 days)
			Voiding pressure	9.00±5.77	8.11±7.39	10.33±4.50
			NVC (%)	43	44	50
			Voiding frequency (time/min)	0.79±0.11	0.82±0.19	0.92±0.07
			Bladder volume (mm3)	1874.47±1452.13	1925.99±1591.44	1594.45±703.36
**Mitsui, 2011 [13]**	9 days after injury	7 weeks after transplantation			N = 9	N = 10, Transplanted (NRP/GRP)
			Voiding pressure		36.1±2.7	27.9±2.0*
			Residual urine		0.16±0.04	0.14±0.08
			Bladder capacity		1.54±0.06	1.53±0.17
			NVC		7.0±1.0	3.4±0.9*
			Bladder weight (mg)		368.3±35.0	289.6±19.6*
**Telmeltas, 2009 [16]**	9 days after injury	28 days after transplantation		N = 5	N = 5	N = 10, Transplanted (NRP/GRP)
			Voiding pressure	24.90±4.03	61.80±9.41	43.40±5.71*
			Residual volume	0.05±0.07	1.61±0.23	0.64±0.12*
			Bladder capacity	0.57±0.13	1.89±0.18	1.64±0.13*
			NVC	0.20±0.44	5.80±2.58	4.10±1.91*
			Baseline pressure	2.15±0.54	8.78±0.55	3.38±0.30*
			Voiding volume	0.56±0.12	0.27±0.05	1.02±0.08*
				N = 5	N = 5	N = 9, Transplanted (BMSC)
			Voiding pressure	24.90±4.03	61.80±9.41	53.10±7.88*
			Residual volume	0.05±0.07	1.61±0.23	0.79±0.20*
			Bladder capacity	0.57±0.13	1.89±0.18	1.70±0.10*
			NVC	0.20±0.44	5.80±2.58	4.90±2.60*
			Baseline pressure	2.15±0.54	8.78±0.55	5.16±1.03*
			Voiding volume	0.56±0.12	0.27±0.05	0.90±0.18*
**Mitsui. 2005 [18]**	9 days after injury	8 weeks after transplantation		N = 6	N = 11	N = 12
			Voiding pressure	19.5±1.0	35.9±1.4	28.2±0.9*
			Residual volume	0.03±0.01	0.24±0.03	0.23±0.09
			Bladder capacity	0.44±0.06	1.07±0.09	1.12±0.12*
			NVC	0	6.7±1.0	3.2±0.8*
			Bladder weight	119.3±6.7	376.8±31.7	279.8±17.8*
**Mitsui, 2005 [14]**	9 days after injury	8 weeks after transplantation		N = 6	N = 10	N = 8
			Voiding pressure	19.5±1.0	33.4±1.2	27.9±2.0*
			Residual volume	0.03±0.01	0.24±0.05	0.14±0.08
			Bladder capacity	0.44±0.06	1.66±0.19	1.53±0.17
			NVC	0	7.2±1.0	3.4±0.9*
			Bladder weight	119.3±6.7	376.8±33.4	289.6±19.6*
**Mitsui, 2003 [19]**	9 days after injury	28 days after transplantation			N = 5	N = 7
			Voiding pressure		39.0±7.4	23.2±4.2*
			Residual volume		3.14±1.84	0.94±0.75*
			Voiding volume		0.94±0.30	1.13±0.22
			Voiding efficiency (%)		27.9±16.6	60.7±19.8*
			NVC (%)		60	57

NVC, non-voiding contraction (No. of episodes/micturition);NRP, neuronal-restricted precursor; GRP, glial-restricted precursor; NBQX, 2,3-dihydroxy-6-nitro-7-sulfamoyl-benzo(f)quinoxaline

*:p<0.05, transplanted group vs. control

### Risk of bias in the included studies

A summary of methodological domain assessment for each subject is detailed in Table 1. Only two studies demonstrated the possibility of bias in blinding of outcome assessment.

**Fig 2:**
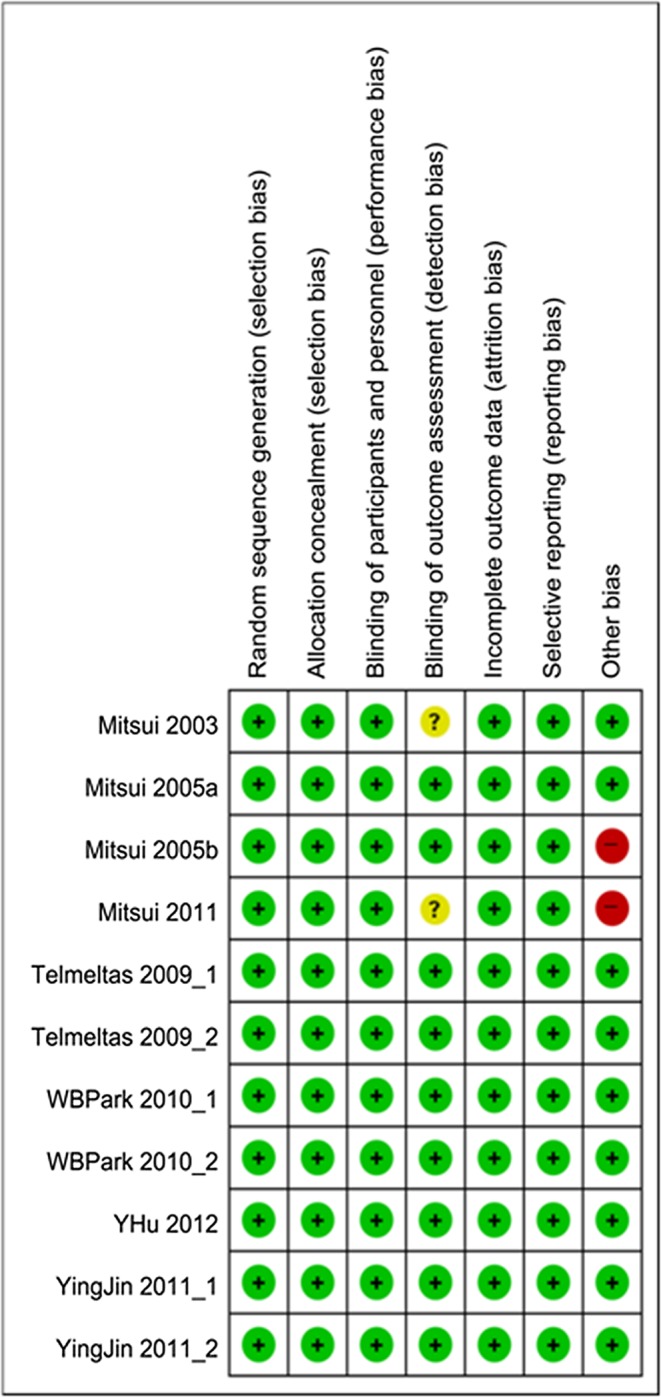
Risk of bias summary. **A review of the author’s judgments about each risk of bias** item for each included study. “+”is “low risk”, “-“is “high risk”, “?” is “unclear”.

These studies did not include the sham model. Overall, the risk of bias was considered to be low (Fig. 2).

### Urodynamic findings

Detailed findings of the urodynamic studies are described in Table 3. Voiding pressure (11 trials), residual urine (9 trials), bladder capacity (10 trials) and NVC (8 trials) were analyzed. Study trials examining bladder weight, baseline pressure, voiding frequency and voiding efficiency were too small for analysis, and therefore those variables were excluded.

**Fig 3:**
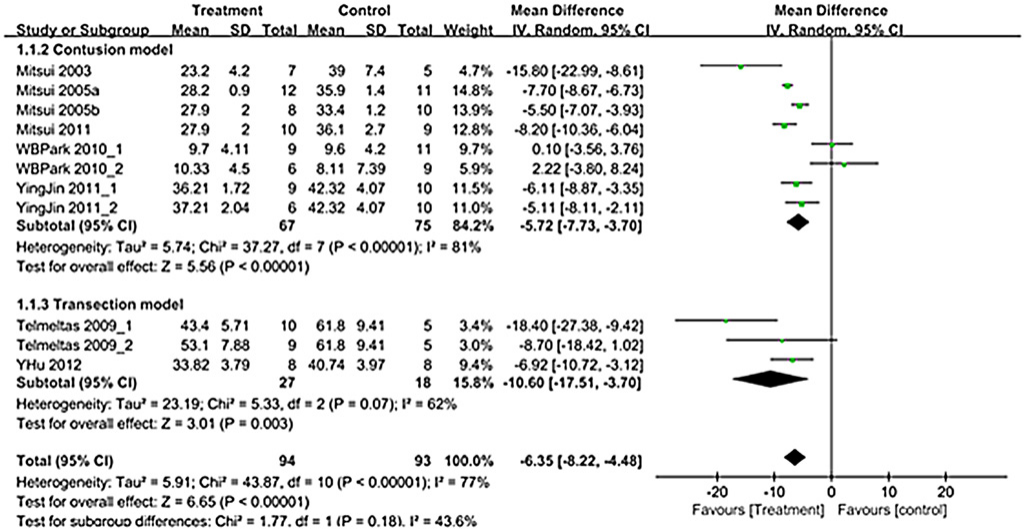
Forest plot diagram showing the effect of stem cell-based cell transplantation on voiding pressure. The black diamond signifies the mean difference is in favor of voiding pressure. The size of each square depends on the weight of each study. All data provided are for continuous outcomes.

A total of 11 trials (n = 187; 94 experimental and 93 controls)[12–19] reported detailed data on voiding pressure. The WMD change of voiding pressure improvement from baseline was -6.35 (95% CI; -8.22, -4.48) (p<0.00001) (Fig. 3). Heterogeneity test showed *p* <0.00001 and Higgins’ I^2^ was 77%. Test of subgroup differences showed *p* = 0.18 and Higgins’ I^2^ was 43.6%.

**Fig 4:**
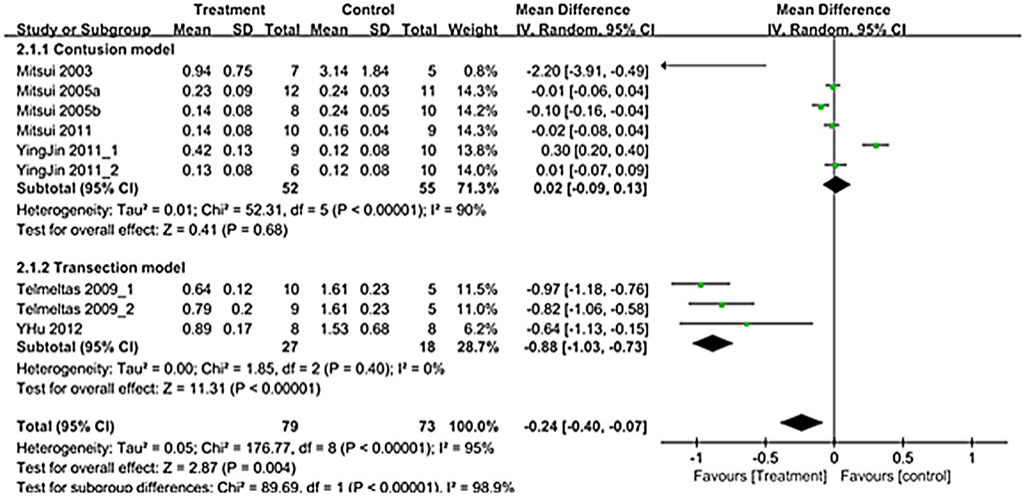
Forest plot diagram showing the effect of stem cell-based cell transplantation on residual urine. The black diamond signifies that the mean difference is in residual urine. The size of each square depends on the weight of each study. All data provided are for continuous outcomes.

A total of 9 trials in seven studies (n = 152; 79 experimental and 73 controls)[12–14,16–19] reported detailed data on residual urine. The WMD change of residual urine improvement from baseline was -0.24 (95% CI; -0.40, -0.07)(p = 0.004)(Fig. 4). Heterogeneity test showed *p* <0.00001 and Higgins’ I^2^ was 95%. Test of subgroup differences showed *p* <0.00001 and Higgins’ I^2^ was 98.9%. In transection model, residual urine showed significant improvement that the WMD change of residual urine in transection model was -0.88 (95% CI; -1.03, -0.73) (p<0.00001) (Fig. 4).

**Fig 5:**
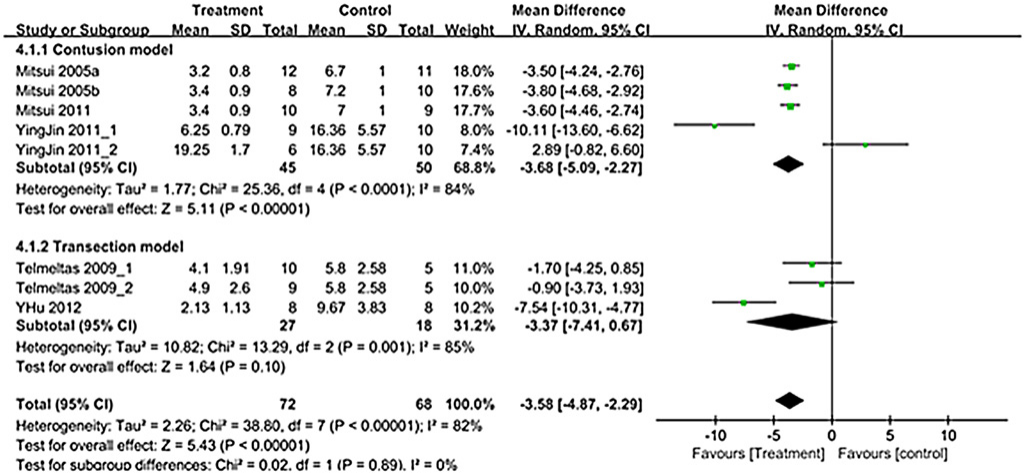
Forest plot diagram showing the effect of stem cell-based cell transplantation on non-voiding contraction (NVC). The black diamond signifies the mean difference is in favor of NVC. The size of each square depends on the weight of each study. All data provided are for continuous outcomes.

A total of 8 trials in six studies (n = 140; 72 experimental and 68 controls) [12–14,16–18] reported detailed data on NVC. The WMD change of NVC improvement from baseline was -3.58 (95% CI; -4.87, -2.29) (p<0.00001) (Fig. 5). Heterogeneity test showed *p* <0.00001 and Higgins’ I^2^ was 82%. Test of subgroup differences showed *p* = 0.89 and Higgins’ I^2^ was 0%. In contusion model, residual urine showed significant improvement that the WMD change of NVC was -3.68 (95% CI; -5.09, -2.27) (p<0.00001) (Fig. 5).

**Fig 6:**
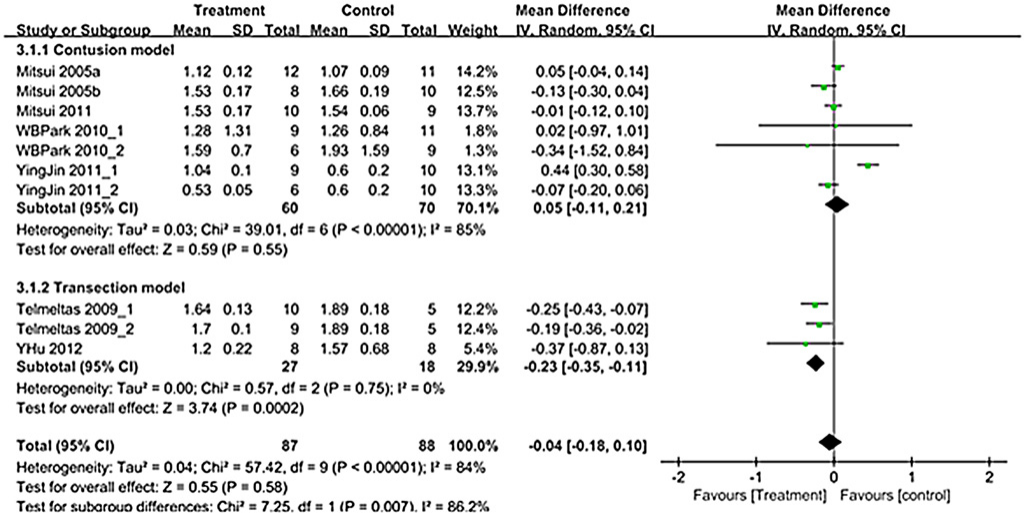
Forest plot diagram showing the effect of stem cell-based cell transplantation on bladder capacity. The black diamond signifies the mean difference is in favor of bladder capacity. The size of each square depends on the weight of each study. All data provided are for continuous outcomes.

A total of 10 trials in seven studies (n = 175; 87 experimental and 88 controls) [12–18] reported detailed data on bladder capacity. Overall mean difference showed -0.04 (95% CI;-0.18, 0.10) (p = 0.58) (Fig. 6). Bladder capacity showed different results according to SCI types. Although contusion model showed no significant improvement after treatment (*p* = 0.55), transection model showed significant improvement after treatment (*p* = 0.0002) (Fig. 6).

### Publication bias

**Fig 7:**
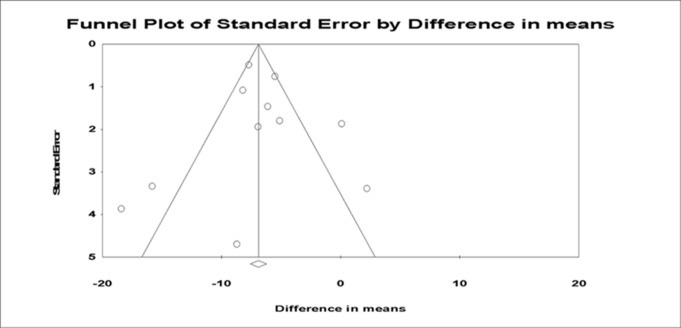
Funnel plot with peusdo 95% confidence limit.

Funnel plot analysis of 11 studies reporting voiding pressure is summarized in Fig. 7. Two studies positioned left of the funnel and two studies lie to the right. Begg and Mazumdar’s correlation was -0.091 (two-tailed p = 0.697). Egger’s regression intercept was -0.230 (two-tailed p = 0.842). Thus, there was no evidence of publication bias in this meta-analysis.

## Discussion

The use of neuroregeneration in stem cell-based cell therapy to correct bladder dysfunction is a logical and promising strategy. Spinobulbospinal pathways regulate and coordinate micturition reflexes. These pathways are located predominately in the dorsal and lateral columns of the spinal cord [20,21]. The contusion injury model, which is the most common SCI model, features the preferential destruction of the dorsal region of the spinal cord. This region is also destroyed in the transection model because injury to this region is necessary to study the spinal cord. The rat model of bladder dysfunction caused by SCI resembles the human condition [6,22,23].

Although complete recovery from SCI is impossible, several studies have demonstrated some functional recovery including limb function and sensory function. To date, very few studies have demonstrated bladder recovery utilizing urodynamic studies including cystometry (Table 3). This is the first study in which a detailed meta-analysis of urodynamic findings relevant to bladder function has been done.

Among the limitations of our study, the most important one is the several heterogeneities including types of SCI models and cell types (Tables 1–3). To control this heterogeneity, sub-analysis regarding the type of SCI model (contusion vs. transection) was done. Concerning the cell types, we did not analyze the subgroup analysis because the nature of the cells was similar and they shared the property of capability of regeneration into neuronal cells. Concerning the transplantation route, all studies adapted direct transplantation into the injured site except for one's study which had adopted the intravenous route. We did not conduct a subgroup analysis for this issue because of the small number of available studies and considering the homing and migration activity of stem cells.

Despite these heterogeneities, the timing of cell transplantation was the same (9 days after SCI), as was the timing of assessment of urodynamics (at least after 28 days following cell transplantation). Moreover, the injured site was similar with the thoracic vertebrae.

SCI weakens voluntary bladder and external urethral sphincter control in rats [21]. The main pathological micturition mechanism for bladder dysfunction following SCI is continuous co-contractions of the bladder and the sphincter leading to inefficient voiding and large residual urine volumes [24]. Continuous high bladder pressure and urine retention in SCI rats leds to complete deterioration of bladder compliance, function, infection and other lower urinary tract complications [25]. Augmented collagen deposition is considered to be an indicator of reduced bladder compliance, consistent with reports of experimental animal models with SCI [20,26]. SCI rats also exhibited frequent urination [7].

Presently, there was a significant improvement of voiding pressure after stem cell-based cell transplantation in the both contusion and transaction models (Figs. 3). Residual urine and bladder capacity improved after treatment only in the transection model, and NVC improved after treatment only in the contusion model (Figs. 4–6).

Improved incidence of NVC during bladder filling has been described in all but two studies [15,19]. The main mechanism for this is that transplanted stem cells inhibit unmediated C-fiber sprouting from bladder afferents, which decreases the C-fiber bladder-to-bladder spinal micturition reflex in SCI rats [18,19].

It is logical to ascribe the late recovery to the parasympathetic system than somatic pathway system. Temeltas et al. [16] reported this issue in their recent study on transplantation of neuronal-glial restricted precursors or neural cells to rats with traumatic SCI [8], suggesting that recovery of function mediated by parasympathetic systems may be more difficult to accomplish than function mediated by somatic pathways [19].

The recovery of a micturition reflex is demonstrating by the descending projections, which diminish sprouting by C-fibers and by the provision of greater descending control over sensory transmission in the NVC [22,23,27,28]. Mitsui et al. [14] reported that the sprouting of primary afferents contributes to bladder dysfunction, specifically NVC, but the more nearly normal immunoreactivity can account for the diminished NVC in neural restricted precursor (NRP)/glial restricted precursor (GRP) rats.

Significant improvements were observed in voiding pressure and residual urine during micturition, which were attributable to alleviating sphincteric dysfunctions. The improvement of NVC and voiding pressure is due to the amelioration of dyssynergia between the bladder and urethral sphincter [19,29]. Attributable improvements in the neuronal circuitry include the central nervous system and the normalization of bladder tone. However, there was no decrease in the bladder capacity in most studies, compared with controls. Such an increase in the bladder capacity is thought to be caused by bladder over-distention during the reflex period by SCI itself [30]. The main mechanism of recovery of voiding function in the two different types of SCI is different. In the contusion model, the main mechanism involves the promotion of partial sparing of descending modulatory pathways that course through the lateral and ventral funiculi to the lumbosacral cord [14,18]. Spinal cord contusion interrupts axons from both ascending and descending tracts. A recent study demonstrated the effects of cell transplantation on general axon growth using neurofilament staining, and on regeneration of the afferent axons and descending serotonergic axons [31]. Voiding pressures were lower after transplantation of NRP/GRP, which indicates the amelioration of dyssynergia between the bladder and urethral sphincter [14,18,19,32]. In the transection model, the main mechanism is the reorganization of synaptic connections in dorsal gray commissure of L3–4, which is responsible for the genesis of the new voiding reflex pathways in SCI rats. Marson [33] and Lee et al. [34] found that neurons in the dorsal gray commissure activated by pudendal or pelvic nerve stimulation might integrate afferent signal neurons within multiple spinal segments.

Cell transplantation for the treatment of SCI has been studied extensively over the last two decades. A wide variety of stem cell-based cell types have been used based on their potential to regenerate myelin, promote and guide axonal growth, bridge the site of injury or differentiate into neuronal or glial cells. Lepore et al. reported that NRP/GRP cells remain viable in the injured spinal cord, migrate outside of the injured region, differentiate into mature central nervous system phenotypes and support neuronal development [35].

Preclinical SCI studies have used neural stem cell/progenitor cells (NSCs/NPCs), umbilical cord blood derived cells, mesenchymal stem cells (MSCs), induced pluripotent cells (iPSCs), Schwann cells and olfactory ensheathing cells. Our enrolled studies measuring bladder dysfunction used NSCs/NPCs [12–14,16,18], MSCs or bone marrow stromal cells [15,17] and embryonic NSCs [19] (Table 1).

In the adult mammalian central nervous system, the genesis of new neurons has been documented, specifically in the subgranular layer of the dentate gyrus of the hippocampus and the subventricular zone (SVZ) of the lateral ventricles [36]. In these regions, adult NPCs self-renew and become multipotent after *in vitro* transplantation into the central nervous system [37,38].

Despite significant advances in transplantation using NPCs, several disadvantages remain. These include inefficient tracking systems and moderate cell survival [39]. In addition, axonal regeneration by endogenous or exogenous NSCs may contribute to the formation of a glial scar [40]. Nonetheless, adult NSCs represent a safe, non-tumorigenic source of trophic support that may have merit for clinical application. Despite an absence of evidence of differentiation into neuronal cell types, several experimental studies have resulted in functional recovery in animals, supporting their potential use in cell therapy in SCI.

Among the enrolled studies, five used NPCs including neural restricted precursors, glial restricted precursors, human glial restricted progenitors, derived astrocytes and transgenically modified fibroblasts [18]. In SCI experimental models, MSCs were used less than NPCs, and MSCs were better in bladder outlet models. The main reason for this tendency is that NSCs/NPCs are considered more trophic and produce more favorable outcomes of neuronal regeneration after direct stem cell transplantation into the injured site.

MSCs were the first type of stem cells used to treat patients and, until recently, were the only stem cell type whose safety had been established [2]. Moreover, MSCs can transdifferentiate into neurons and glial cells [41], and can act in the physical guidance of neurofilament outgrowth in SCI [42]. However, among the enrolled studies, only two included human MSCs and two included bone marrow stromal cells [15,17] (Table 1). To date, most clinical trials of SCI have involved MSC-based cell therapy because of their relative ease of acquisition and safety [10]. To date, the role of MSCs in SCI is limited to the reduction of the inflammatory process within the injured spinal cord. Park et al. [15] could not demonstrate direct neuronal regeneration after transplantation of MSCs. However, clinical trials have shown limited bladder recovery using simple questionnaires and none included urodynamic studies.

Limitations in our study included heterogeneity. Lack of reproducibility resulting partly from differences in SCI models and treatment methods is the most critical issue. To date, no standard tool has been developed to check study quality, which makes meta-analysis of experimental research difficult.

## Conclusions

The present meta-analysis and systematic review regarding bladder recovery after stem cell-based cell therapy in SCI demonstrates significant improvements in voiding pressure, residual urine and NVC. Considering the large volume of research on stem cell therapy in SCI preclinical studies, additional studies are necessary for improved understanding of bladder recovery.

## Supporting Information

S1 PRISMA Checklist (DOC)
